# The genomic profiling and MAMLD1 expression in human and canines with Cushing’s disease

**DOI:** 10.1186/s12902-021-00845-z

**Published:** 2021-09-13

**Authors:** Andrew Wang, Stewart G. Neill, Scott Newman, Marianna A. Tryfonidou, Adriana Ioachimescu, Michael R. Rossi, Björn P. Meij, Nelson M. Oyesiku

**Affiliations:** 1 David Geffen School of Medicine, University of California, Los Angeles, Los Angeles, CA USA; 2 College of Medicine, Charles R. Drew University of Medicine and Science, Los Angeles, CA USA; 3 Department of Pathology and Laboratory Medicine, Emory University School of Medicine, Atlanta, GA USA; 4 Department of Computational Biology, St. Jude Children’s Research Hospital, Anchorage, TN USA; 5 Department of Clinical Sciences of Companion Animals, Faculty of Veterinary Medicine, Utrecht University, Utrecht, Netherlands; 6 Department of Neurosurgery, Emory University School of Medicine, Atlanta, GA USA; 7 Department of Medicine, Emory University School of Medicine, Atlanta, GA USA; 8 Department of Genetics and Genomic Sciences, Icahn School of Medicine at Mount Sinai, New York City, NY USA

**Keywords:** Cushing’s disease, Pituitary adenomas, Dog and man sequencing, MAMLD1

## Abstract

**Background:**

Cushing’s disease (CD) is defined as hypercortisolemia caused by adrenocorticotropic hormone (ACTH)-secreting pituitary adenomas (corticotroph PA) that afflicts humans and dogs. In order to map common aberrant genomic features of CD between humans and dogs, we performed genomic sequencing and immunostaining on corticotroph PA.

**Methods:**

For inclusion, humans and dog were diagnosed with CD. Whole exome sequencing (WES) was conducted on 6 human corticotroph PA. Transcriptome RNA-Seq was performed on 6 human and 7 dog corticotroph PA. Immunohistochemistry (IHC) was complete on 31 human corticotroph PA. Corticotroph PA were compared with normal tissue and between species analysis were also performed.

**Results:**

Eight genes (MAMLD1, MNX1, RASEF, TBX19, BIRC5, TK1, GLDC, FAM131B) were significantly (*P* < 0.05) overexpressed across human and canine corticotroph PA. IHC revealed MAMLD1 to be positively (3+) expressed in the nucleus of ACTH-secreting tumor cells of human corticotroph PA (22/31, 70.9%), but absent in healthy human pituitary glands.

**Conclusions:**

In this small exploratory cohort, we provide the first preliminary insights into profiling the genomic characterizations of human and dog corticotroph PA with respect to MAMLD1 overexpression, a finding of potential direct impact to CD microadenoma diagnosis. Our study also offers a rationale for potential use of the canine model in development of precision therapeutics.

**Supplementary Information:**

The online version contains supplementary material available at 10.1186/s12902-021-00845-z.

## Background

Corticotrope pituitary adenomas (PA) are benign tumors of the anterior pituitary gland that secrete excessive amounts of adrenocorticotropin hormone (ACTH), causing Cushing’s disease (CD) [1]. The precursor polyprotein to ACTH, proopiomelanocortin (POMC), is synthesized within residential anterior pituitary corticotropic cells [2]. When ACTH is secreted, zona fasciculata cells within the adrenal cortex release cortisol, which exerts diverse physiological systemic effects [3, 4]. Mortality is increased up to 5 times in CD compared with the general population, but significantly improves after achievement of normocortisolemia by treatment [5, 6].

CD treatment options are diverse. Surgery is the first choice treatment and removal of the tumor results in remission in 75–90% of patients with a 35% biochemical recurrence [4, 7–10]. The remainder have either small tumors not identified at surgery or nonresectable tumors that invade the cavernous sinus [11]. Other options include radiotherapy and bilateral adrenalectomy, however these procedures have risks of permanent cortisol and aldosterone deficiency along with potentiation of aggressive tumor growth and inevasible behavior (Corticotroph Tumor Progression) [7]. Finally, while medical treatment options have expanded to include somatostatin receptor ligands, cortisol synthesis inhibitors, glucocorticoid receptor blockers, and dopamine agonists, long-term safety data continues to be lacking [12–15]. Taken together, many of the available compounds target the adrenal gland or suppress ACTH secretion, but not the source of the secreting PA, therefore there is a strong need for further research on this topic.

Because CD is a rare tumor (incidence of 4:1,000,000 [16, 17]), research has yielded a limited number of dysfunctional genes for diagnostic or treatment purposes. While genes (POMC, t-box transcription factor 19 (TBX19), ubiquitin carboxyl-terminal hydrolase 8 (USP8), ubiquitin carboxyl-terminal hydrolase 48 (USP48) and cyclin-dependent kinase 5 and abelson murine leukemia viral oncogene homolog 1 enzyme substrate 1 (CABLES1)) associated to PA have been identified [2, 18–27], preclinical studies suggest that only USP8 PA mutation and TBX19 expression may respond to epidermal growth factor receptor (EGFR) inhibition and cyclin-dependent kinase (CDK) inhibitors, respectively [18–21]. With limited results, a broader catalogue of PA mutations are needed to explore a wider variety of treatment diagnosis options.

Dogs may be viable models for targeted interventions in CD. Dogs share an 84% genetic overlap with humans, and they naturally develop Cushing’s disease from the intermediate pituitary lobe with an incidence of 1500:1,000,000 and recurrence after hypophysectomy of 27%, analogous to humans [28–32]. A canine disease model has advantages over transgenic mouse models, particularly since dogs share similar environmental exposures and stressors to humans [29]. However, while TBX19 expression were demonstrated in dog PA, USP8 mutations were not identified as frequently mutant [33]. This prompted us to investigate a more complete catalogue of genomic aberrations that may lead to dysfunctional protein expression observed in CD and could potentially lead to the development of targeted pharmaceutical interventions.

Using both human and dog corticotroph PA, our objective was to describe the common aberrant genomic features of CD between humans and dogs. We profiled genetic expression using whole transcriptome sequencing of both species. Whole exome sequencing (WES) was also performed on the human corticotroph PA and an identified target was confirmed by immunohistochemistry when compared to normal human pituitary tissue. Across experimentation, corticotroph PA were compared with normal tissue, and when possible between species analysis were also performed. Furthermore, although genetic studies have been previously conducted in humans with CD [15, 18, 19, 24, 34–37], our results provide a broad overview of the genetic profiles between human and dog corticotroph PA.

## Methods

For eligibility, human and dogs were required to have a CD diagnosis. Genomic sequencing and immunostaining were performed on corticotroph PA. Across experimentation, corticotroph PA were compared with normal tissue, and when possible between species analysis were also performed.

### Human cohort

Thirty-seven CD African American patients (average 43 +/− 12 years old) with intermediate anterior sized PA (avg lateral 1.16 +/− 0.44 cm x AP 0.88 +/− 0.29 cm x cranial 0.90 +/− 0.49 cm) were recruited through the Department of Neurosurgery at Emory University Hospital. In all cases, the diagnosis of Cushing’s disease entailed the following steps: 1. two or more abnormal screening tests (including lack of suppression to low-dose dexamethasone, higher than normal urinary free cortisol levels and/or higher than normal late-night salivary cortisol levels), 2. ACTH-dependent hypercortisolism (i.e. high or high-normal ACTH level) and 3. localization tests supporting autonomous pituitary ACTH secretion (cortisol suppression by more than 50% or below 5 mcg/dL after to high-dose dexamethasone and/or ACTH stimulation after CRH test). In addition, depending on pituitary MRI findings, selected patients underwent petrosal sinus sampling which indicated a baseline central-to-periphery ACTH ratio > 2 and/or post-CRH ratio > 3). For all cases, pathology reports showed immunopositive staining for ACTH, and negative immunostaining for tumor protein p53 (TP53), follicle-stimulating hormone (FSH), thyroid stimulating hormone (TSH), luteinizing hormone (LH), growth hormone (GH), and E3 ubiquitin protein-ligase (MIB-1) proliferation index < 3%. During microsurgical transsphenoidal hypophysectomy, the PA tissue was removed with no complications.

In total, 39 human specimens were utilized (37 corticotroph PA, 2 normals). For WES and RNA-seq, frozen specimens were available from 6 African American patients (1 male, 5 females) and normal human pituitary specimens from 1 male (ND01199–09) and 1 female (ND01218–06) were purchased from the Coriell Biorepository (Camden, NJ). For IHC, paraffin specimens were available from 31 African American patients with 1 normal anterior pituitary tissue.”

### Dog cohort

CD Dogs from canine client owners were recruited through the Department of Clinical Sciences of Companion Animals at the Utrecht University, the Netherlands. Suspicion of hypercortisolism was raised based on the characteristic clinical signs including polyphagia, polydipsia/polyuria, skin atrophy, thin hair coat, calcinosis cutis, truncal obesity (pot belly), depression, and exercise intolerance. Serum chemistry showed typical changes: alkaline phosphatase, alanine aminotransferase and total cholesterol were commonly increased in canine Cushing’s disease.

Preliminary diagnosis of pituitary-dependent hypercortisolism was based on increased urinary corticoid-to-creatinine ratios (UCCRs) in the first two morning urinary samples collected at home and more than 50% suppression of the UCCR in the third urine sample in the oral high dose (0.1 mg/kg) dexamethasone suppression test [32]. CD was further confirmed by measurement of elevated plasma ACTH concentration, visualization of symmetrically enlarged adrenal glands by ultrasonography, and visualization of pituitary gland enlargement with computed tomography (CT) or MRI. During microsurgical transsphenoidal hypophysectomy in client-owned dogs, the PA tissue was removed with no complications. Immediately after collection, specimens of pars distalis PA tissue were fixed in 4% neutral buffered formaldehyde, embedded in paraffin, and consecutive sections were used for histology and immunohistochemistry for ACTH, α-melanocyte-stimulating hormone (MSH), and GH [32]. Representative adenoma tissue samples were snap-frozen and stored in liquid nitrogen until analysis after histology confirmed a basophilic adenoma with ACTH immunostaining.

Pituitary specimens from 6 healthy beagle dogs and 7 client-owned dogs with CD that underwent hypophysectomy were sequenced. The cohort of client-owned dog patients included different breeds (i.e., Jack Russell Terrier, Scottish Shepherd, English Springer Spaniel, Mixed breed, French Bulldog, Chesapeake Bay Retriever, and American Staffordshire Terrier), 3 female (of which two castrated) and 4 male (of which one castrated) dogs, with a median age of 8.2 years (range 5.6–10.7 years). All owners consented to the hypophysectomy as treatment for CD. As control tissue, anterior lobes of normal pituitary glands were obtained from 6 healthy Beagle dogs euthanized in unrelated experiments (all female; age range 1.6–1.8 years).

### Nucleic acid extraction and sequencing

DNA and RNA were extracted from 6 intermediate sized frozen human PA tissues using E.Z.N.A. Ⓡ kits (Omega Bio-tek, Norcross, GA). RNA was extracted from the canine healthy and tumor pituitary samples using the miRCURY cell and plant RNA isolation kit according to the manufacturer’s instruction including an on-column DNAse digestion (Qiagen, RNAse free DNAse, 79,254). Specimen quantity and quality were assessed using Qubit and Agilent Bioanalyzer.

### Human exome sequence analysis

Human genomic DNA was extracted from fresh-frozen PA and normal anterior pituitary as described above. Whole human exome libraries were prepared using the Agilent SureSelect Human All Exon (version 5) per manufacturer’s protocols. Libraries were paired-end sequenced at 50x coverage using and Illumina HiSeq 2000. FASTQ files were aligned to the human hg19 reference genome using BWA 0.7.5.a and duplicate reads were removed with Picard tools (Version 1.1.1) [32, 38]. De-duplicated aligned (BAM) files were used for copy number estimation using Control-FREEC with recommended settings for exome sequencing [39]. Normalized FREEC output values were segmented with DNACopy [40]. Copy number assessments for 6 PA samples were matched to one normal. Human PA specimen 1, had a matched normal sample and mutations were called using Varscan2 in somatic mode under standard parameters [41]. Mutations in the other 5 samples were called using Varscan2 without a matched normal. Human PA specimen 1 was also analyzed with Mutect (version 1.1.4) using standard parameters following probabilistic indel realignment and base recalibration using GATK (version 3.3.0) [42]. GISTIC-like analysis using cghMCR were used to identify potential regions of focal gain and loss [43]. Identified single nucleotide variants (SNV) were compared to somatic variants in the COSMIC database (version 81) and gnomAD database [38, 44]. All predicted mutations were annotated using ANNOVAR [45].

### RNA sequence analysis

Total RNA was processed using the Illumina TruSeq RNA kit and paired-end sequenced (2 × 75) at 100 M reads per specimen on a HiSeq 2000 instrument. FASTQ files were aligned to the hg19 human or CanFam3 reference genomes using Tophat 2.0.1 using standard parameters [46]. RefSeq, CanFam3 and Ensembl transcripts were quantified using Cufflinks (version 2.0.1) [47]. Human RefSeq transcripts were also quantified using HTSeq (version 0.6.1) [48]. Mutations were called in the canine and human samples using Varscan2 as described above. Identification of differentially expressed genes (> 2 fold change) between tumor and normal tissue by simple hierarchical clustering of normalized fragments per kilobase of transcript per million mapped reads (FPKM) values were assessed separately for human and dog Cushing samples and dichotomized into over or under gene expression. With the feasible utilization of IHC for conformational protein upregulation, only shared overexpressed genes were analyzed.

### Immunohistochemistry

Immunostaining was performed on 5 μm formalin fixed paraffin embedded (FFPE) sections of 31 corticotroph PA samples and 1 normal anterior pituitary tissue. Sections were deparaffinized in xylene and tissue was hydrated by a descending ethanol sequence. After rehydration, sections were incubated with 3% H_2_O_2_ to inactivate endogenous peroxidases and blocked with 1% BSA for 10 min. Due to limited tissue quantity, a random choice generator (SOCR) selected four of the eight cross-species significantly overexpressed genes (stained - TBX19, RASEF, MAMLD1, MNX1; non-stained - BIRC5, TK1, GLDC, FAM131B) as well as POMC, USP8, and ubiquitin carboxyl-terminal hydrolase 48 (USP48) were selected from reference studies [2, 18, 19, 49]. In total, seven genes were selected for staining. Antibodies against TBX19 (Sigma Aldrich, HPA072686, rabbit 1:2000), MAMLD1 (Sigma Aldrich, HPA003923, rabbit 1:250), MNX1 (Sigma Aldrich, ABN174, rabbit 1:500), RASEF (Sigma Aldrich, WH0158158M1, mouse 1:200), USP8 (Sigma Aldrich, HPA004869, rabbit 1:100), USP48 (Sigma Aldrich, HPA030046, rabbit 1:100), and POMC (Abcam, AB210605, rabbit 1:8000) were used. The antibodies were then incubated at 4 °C overnight and visualized with diaminobenzidine. After staining, 2 tissues were fully exhausted. Image analysis was performed under a light microscope at × 400 magnification. Immunopositivity was graded by two independent reviewers (AW, SGN) with a 0 to 3+ scoring system:

- (0) no immunostaining.
- (1) mild positive: weak immunostaining of less than 30% of tumor cells.
- (2) positive: complete membranous staining and either uniform or weak in at least 50% of tumor cells.
- (3+) strong positive: uniform intense nuclear and/or cytoplasmic staining in at least 80% of tumor cells.

Grading disagreements were reexamined and reassessed collectively by the same reviewers.

### Statistical analysis

Differentially expressed transcripts were converted to normalized fragments per kilobase million (FPKM) values using tools described above [47, 48]. Average log transformed FPKM values for both humans and dog transcripts are expressed with standard deviations. Comparisons of each subset of genes between ACTH secreting PA and normal pituitary tissues across either the human or dog groups were considered statistically significant when *P* < 0.05 in a one-tail Student T-test (i.e. overexpression tail). Immunohistochemistry results were expressed as proportions, mean, standard deviations (SD) and median. Scoring agreements were expressed as a weighted Cohen’s Kappa coefficient. With no censored or missing data, a sensitivity analysis were performed between stained and non-stained genes. Stata/IC (v15.0; StataCorp LP, College Station, TX, USA) statistical software was used for all analyses.

## Results

### Whole exome sequencing

To identify recurrent somatic mutations and copy number abnormalities in corticotroph PA, whole-exome sequencing was performed in human PA.

No specimens with mutations of USP8 were identified. Two human specimens with USP48 (NM_032236) SNV were found. Human PA specimen 1 had a USP48 c.1245 G > A, p.M415I (COSM904151) SNV and specimen 6 had a USP48 c.1243 A > G, p.M415V (COSM25000) SNV.

Two human PA (3 and 4) had guanine nucleotide-binding protein G(q) subunit alpha (GNAQ) c.286A > T, p.T96S substitutions.

**Fig. 1:**
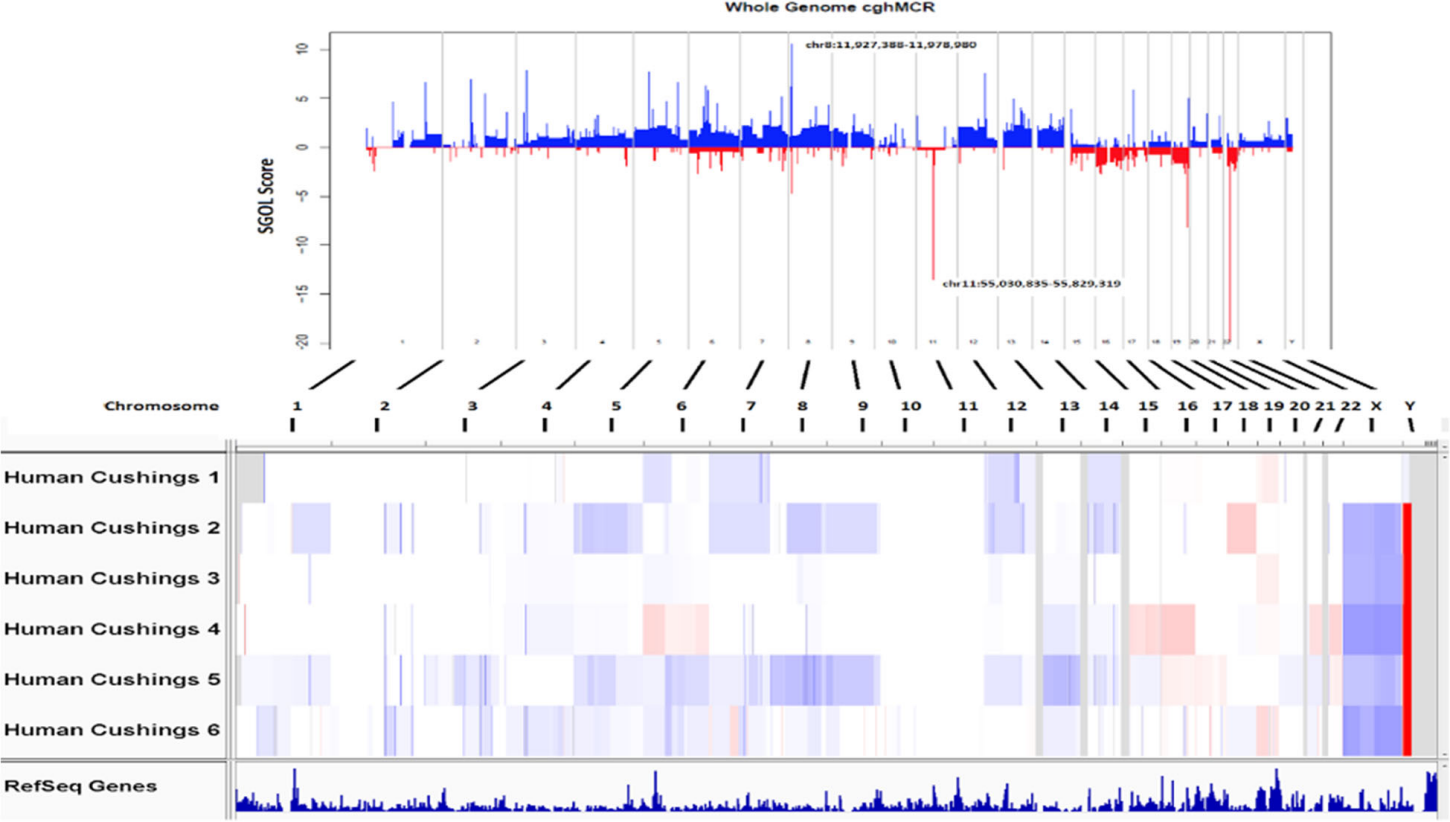
Copy number variation of somatic mutations in corticotroph pituitary adenomas (PA) causing Cushing’s disease. Whole Exome Sequencing of 6 human PA specimens. Copy number gains (blue) and losses (red) are shown as a GISTIC profile (top) and for individual specimens. Prominent regions of focal gain (chr8:11,927,388-11,978,980_hg19) and loss (chr11:55,030,835-55,829,319_hg19) highlighted in the GISTIC profile represent regions of benign copy number variation. Focal gains or losses in 6 human PA specimens were not identified. Gain of whole chromosomes 5, 7, 8, 9, 12, 13 and 14 and loss of chromosome 19 were recurrent copy number abnormalities

Copy number analysis using the whole exome sequencing data was also performed to ascertain recurrent gains of whole chromosomes or chromosome arms (Fig. 1). Human PA specimens 2 and 5 had similar patterns with gain of 1q, 5, 7, 8q, 9, 12, 13 and 14. Specimen 1 shared gain of 7, 12 and 14 with specimens 2 and 5. Loss of 19 was shared by specimens 1, 3 and 6. No focal gains or losses enriched in PA in non-benign copy number variations were observed.

### RNA-sequencing

Gene expression between Cushing’s tumor and normal tissue was assessed in human and dogs.

**Fig. 2:**
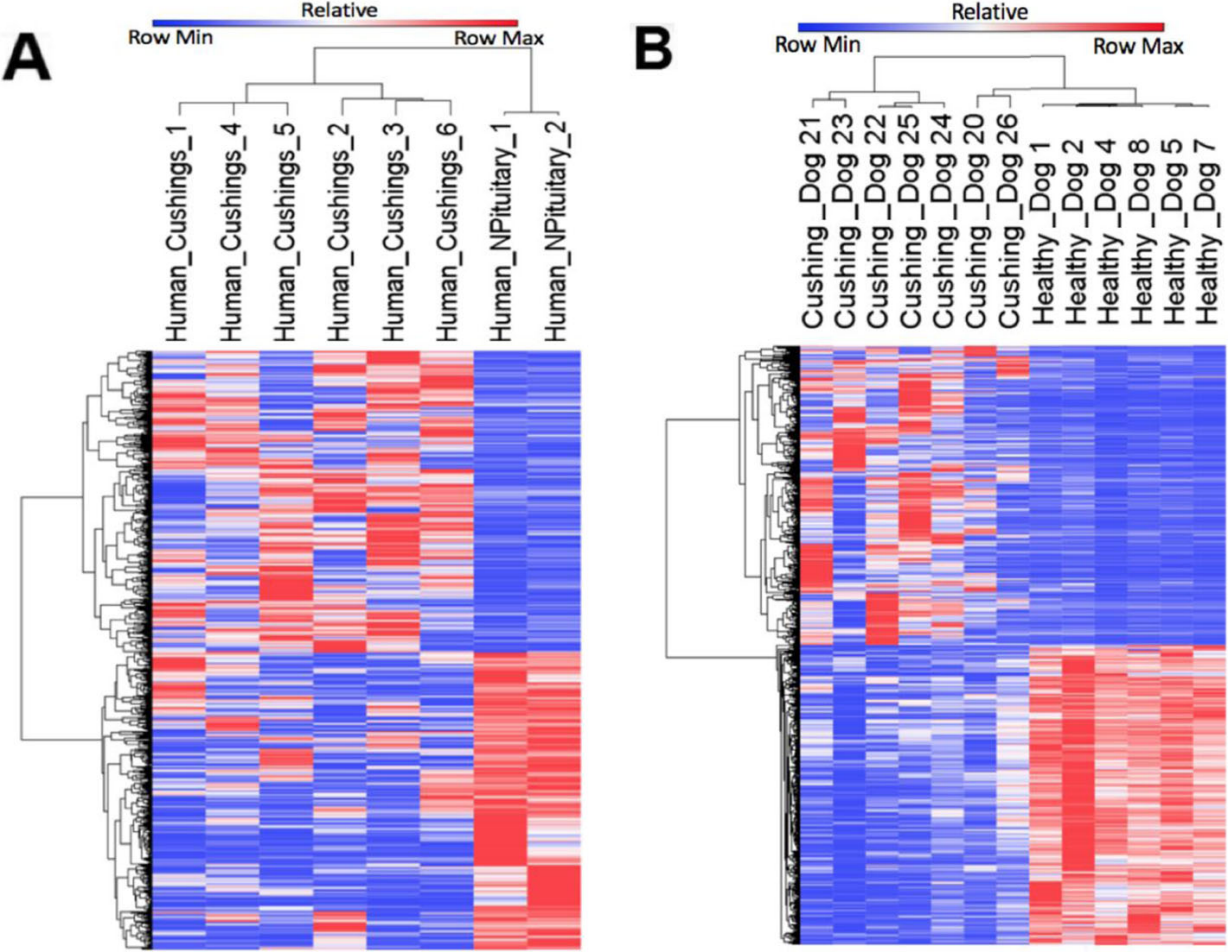
Unsupervised hierarchical clustering of differentially expressed genes in corticotroph pituitary adenoma (PA) vs normal pituitary in dog and human. Normalized RNA-Seq FPKM values of genes were subject to unsupervised hierarchical clustering comparing 6 human PA to 2 normal PA (A) and 7 dog PA to 6 normal dog PA (B). Differential gene expression profiles clearly separated the PA specimens from normal pituitary specimens for dogs and humans

Unsupervised hierarchical clustering showed the normal pituitary specimens clustered separately from the PA specimens for both species (Fig. 2). Heterogeneity of gene expression was found in individual PA specimens in both species, and a subset of genes that were over or under expressed relative to normal pituitary in both species organisms clustered (Fig. 2).

**Fig. 3:**
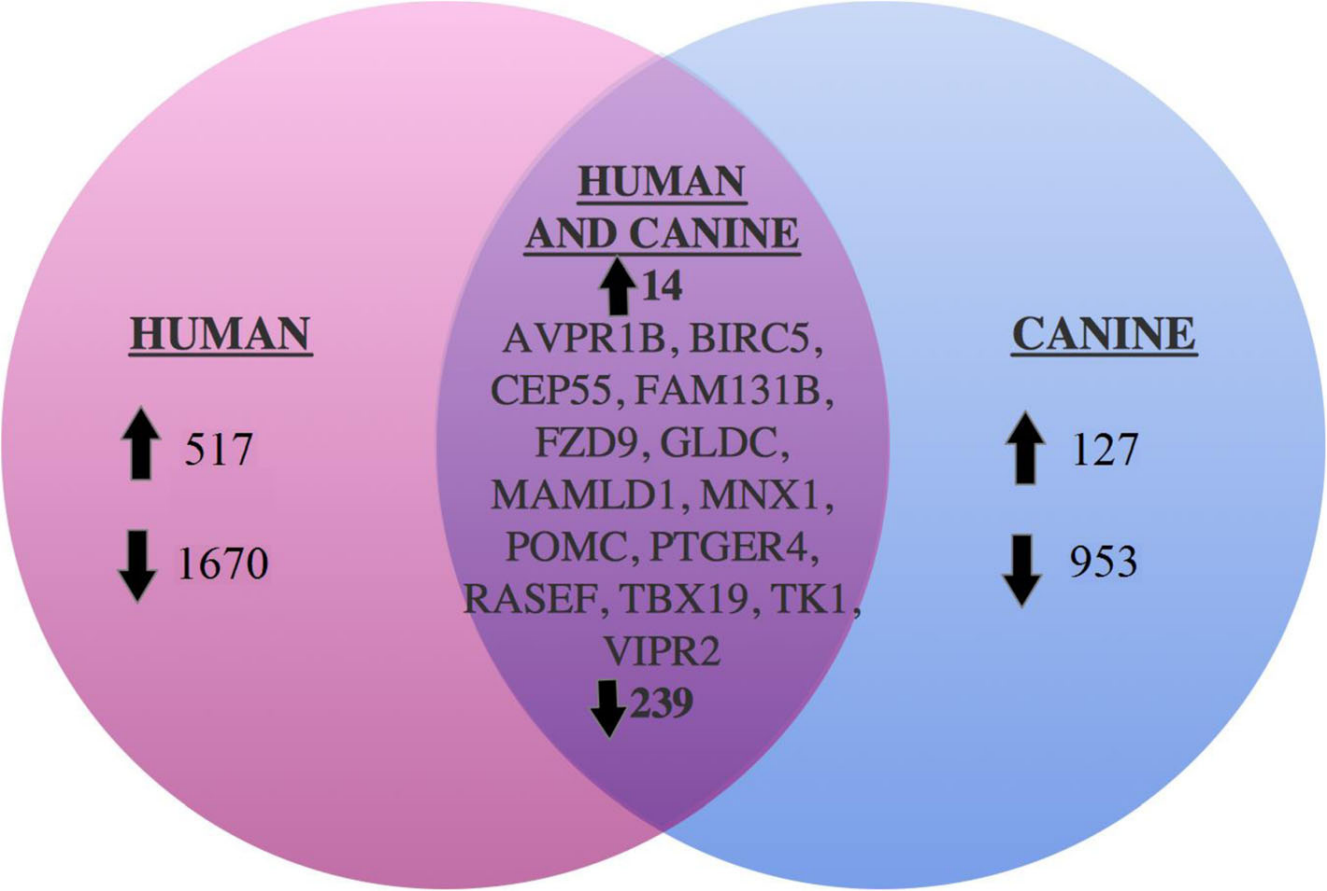
Shared gene expression changes in humans and dogs with Cushing’s disease. Genes were grouped by overexpression (> 2-fold compared to normal) and underexpression (< 2-fold compared to normal) of normalized RNA-Seq FPKM levels of human (red) and dog (blue) specimens. 517 overexpressed and 1670 underexpressed human genes, and 127 overexpressed and 953 underexpressed dog genes were identified. Overlap of the 2 datasets demonstrated 14 overexpressed and 239 underexpressed genes between dog and man. The 14 commonly overexpressed genes were: *AVPR1B, BIRC5, CEP55, FAM131B, FZD9, GLDC, MAMLD1, MNX1, POMC, PTGER4, RASEF, TBX19, TK1,* and *VIPR2*

Differential gene expression showed 517 overexpressed and 1670 underexpressed genes in human PA, and 127 overexpressed and 953 underexpressed genes in dog PA relative to normal pituitary tissues (Fig. 3). Intersection of the human and dog gene lists showed 14 overexpressed genes and 239 under expressed genes shared between species. The 14 highly expressed genes were: POMC, arginine vasopressin receptor 1B (AVPR1B), baculoviral inhibitor of apoptosis repeat-containing 5 (BIRC5), centrosomal protein of 55 kDa (CEP55), family with sequence similarity 131 member B (FAM131B), frizzled-9 (FZD9), glycine decarboxylase (GLDC), mastermind like domain containing 1 (MAMLD1), motor neuron and pancreas homeobox 1 (MNX1), prostaglandin E2 receptor 4 (PTGER4), RAS and EF-hand domain-containing protein (RASEF), t-box transcription factor (TBX19), thymidine kinase 1 (TK1) and vasoactive intestinal peptide receptor 2 (VIPR2).

**Fig. 4:**
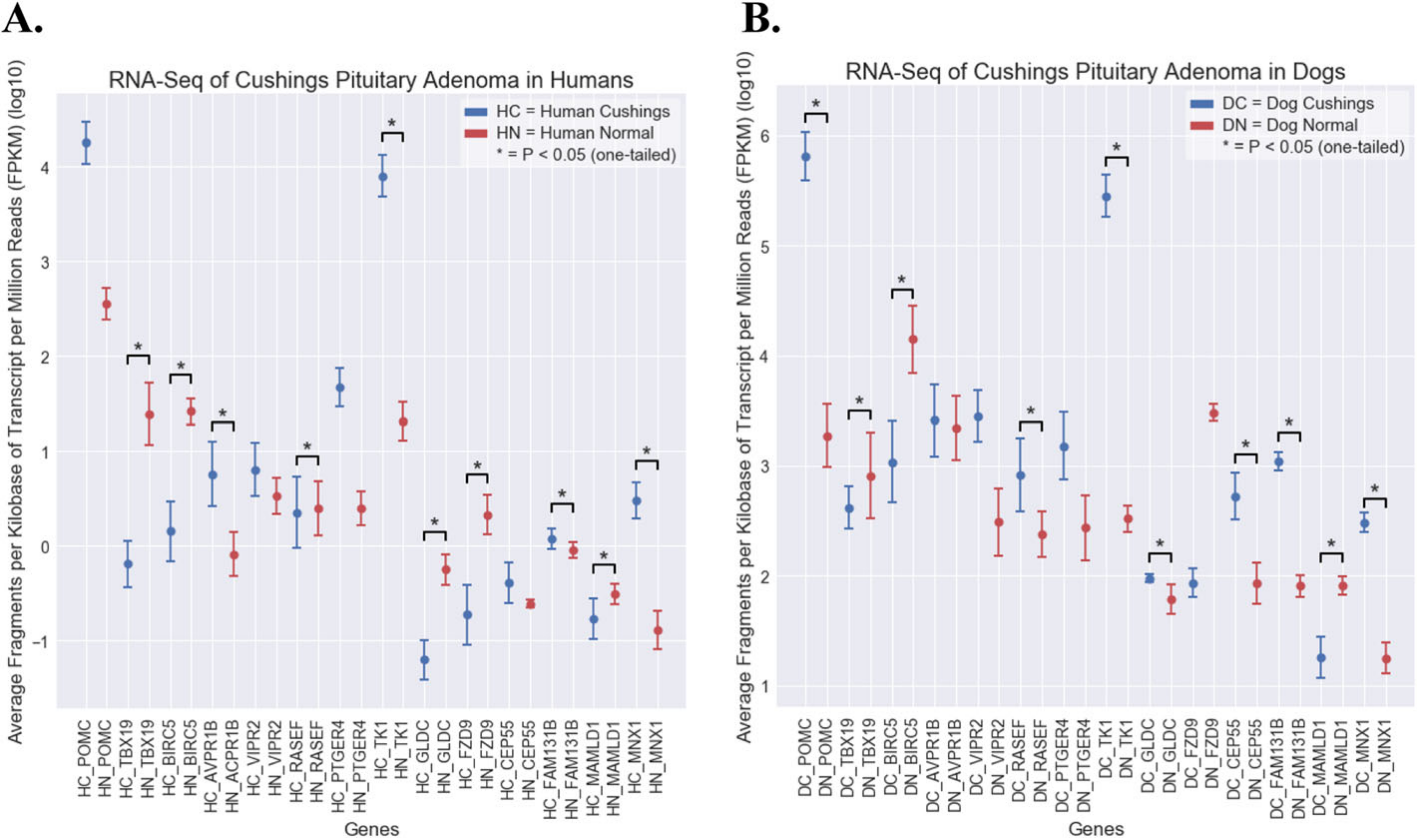
Boxplot comparison of overexpressed genes in humans and dogs. Average comparisons and standard deviations are shown for human corticotroph pituitary adenomas (HC PA, *n* = 6) and human normal pituitary (HN, *n* = 2). Median comparisons and standard deviations are shown for dog corticotroph pituitary adenomas (DC PA, *n* = 7) and dog normal pituitary (DN, n = 6). Normalized FPKM levels for 14 overexpressed genes in corticotroph PA (red) and normal pituitary (blue) are shown for humans (**A**), and dogs (**B**). MAMLD1, MNX1, RASEF, TBX19, BIRC5, TK1, GLDC, and FAM131B were significantly overexpressed in corticotroph PA as compared to normal pituitary (*P* < 0.05) in both humans and dogs. AVPR1B and FZD9 were significantly (P < 0.05) overexpressed in only human PA. POMC, and CEP55 were significantly (P < 0.05) overexpressed in only dog PA

Gene level summaries were performed for the 14 highly expressed genes, comparing PA to normal pituitary (Fig. 4) (SP Fig. 1, 2 and 3). Human samples revealed significant (*P* < 0.05) results for 10 genes: TBX19, BIRC5, AVPR1B, RASEF, TK1, GLDC, FZD9, FAM131B, MAMLD1 and MNX1. In dogs, 10 genes: POMC, TBX19, BIRC5, RASEF, TK1, GLDC, CEP55, FAM131B, MAMLD1 and MNX1 yielded significant results (P < 0.05). Eight genes: MAMLD1, MNX1, RASEF, TBX19, BIRC5, TK1, GLDC, and FAM131B were significant (*P* < 0.05) in PA from both species.

### Immunohistochemistry

**Fig. 5:**
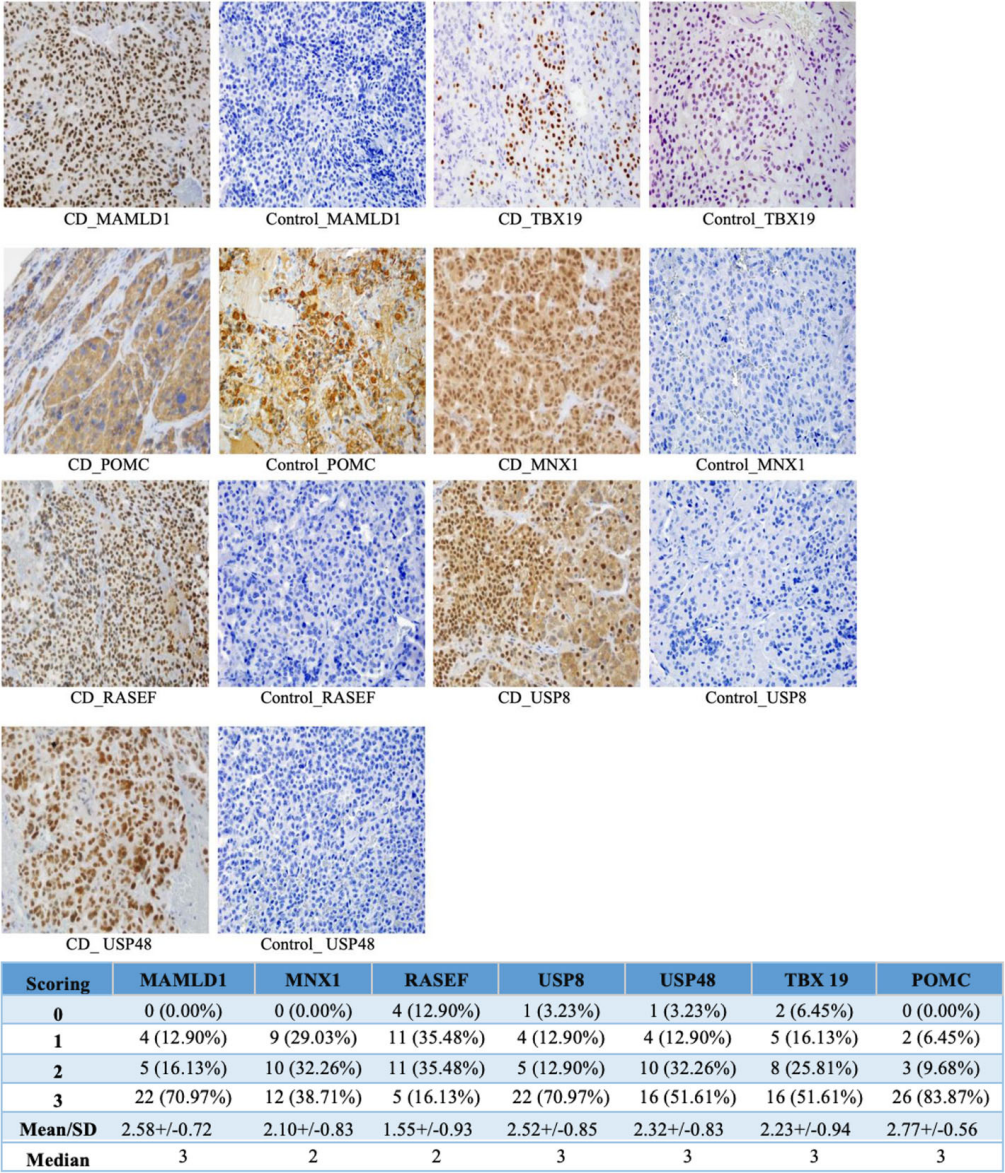
Immunohistochemistry for proteins suspected to be overexpressed in Cushing’s disease. Thirty one human corticotroph pituitary adenomas (PA) were sectioned, stained, and scored (0 to 3+). Respective 3+ PA and negative control normal are presented. POMC (26/31, 83.9%), MAMLD1 (22/31, 70.9%), USP8 (22/31, 70.9%), TBX19 (16/31 50.9%), USP48 (16/31, 50.9%) demonstrated strong (3+) staining. MNX1 (12/31, 38.7%) and RASEF (5/31, 16.2%) displayed less stronger (< 3) staining

To examine the association between significantly expressed genes and protein translation, immunohistochemistry was performed on four of the eight significant genes in human PA (Fig. 5).

Consistently strong (3+) staining was observed for POMC (26/31, 2.77+/− 0.56), MAMLD1 (22/31, 2.58+/− 0.72), USP8 (22/31, 2.52+/− 0.85), TBX19 (16/31, 2.23+/− 0.94), USP48 (16/31, 2.32+/− 0.83) and MNX1 (12/31, 2.10+/− 0.83) (SP Table. 1). RASEF (1.55+/− 0.93) displayed weak staining (< 3). Nuclear staining was observed for TBX19, MAMLD1, RASEF, and USP48. POMC staining was cytoplasmic. MNX1 and USP8 staining was nuclear and cytoplasmic. Specimens with high (3+) MAMLD1 expression shared strong (3+) staining in POMC (21/22, 95.5%) and USP8 (20/22, 90.9%). USP48 (13/22 59.1%), TBX19 (12/22, 54.5%), MNX1 (11/22, 50.0%), and RASEF (5/22, 22.7%) showed limited high coexpressions among high MAMLD1 specimens (SP Fig. 1). While weak (1+) TBX19 and POMC staining was observed in normal anterior pituitary, no staining for MAMDL1, MNX1, RASEF, USP8 and USP48 was found (SP Table. 1).

The strength of agreement between reviewers was almost perfect (k = 0.87). Sensitivity analysis between stained and non-stained genes outcomes were not statistically significant (*P* > 0.05).

## Discussion

Our WES provides the first broad overview of RNA-seq gene expression profiles of PA in dog and man with CD. Mutations in USP8 as the main driver and Guanine nucleotide-binding protein G(s) subunit alpha isoforms short (GNAS) have been implicated in PA tumorigenesis [18, 19, 34].

While USP8 even by manual inspection of aligned DNA and RNA sequencing reads were not found, two separate non-synonymous changes were found at codon 415 of USP48 (p.M415I and p.M415V). Both have been reported as somatic variants in recent Cushing’s PA and in the COSMIC database in cancers arising from the urinary tract and prostate [22]. These variants were unlikely to be either common or rare population variants as they were absent from the gnomAD database. In these variants, methionine 415 is a conserved residue falling within the Peptidase C19 ubiquitinyl hydrolase domain of USP48 and bioinformatic predictions using Polyphen 2 suggested that both changes may affected protein function.

Two specimens had Guanine nucleotide-binding protein G(q) subunit alpha q polypeptide (GNAQ) p.T96S SNVs instead of GNAS mutations. T96S has been reported in 20 COSMIC samples (COSM404628) - predominantly large intestine and skin - and may represent a third hotspot within the protein additional to the well-characterized Q209 and R183 positions. There have been more recent studies that have shown that no significantly recurrent mutations are identified by WES in PA [27, 50]. Combined with our results, these discrepancy illustrates that PA, although rare, may also be a heterogeneous disease from the genetic perspective. Larger cohorts are needed to determine if mutations or copy number abnormalities can be used to stratify and target therapies for PA.

Along with TBX19, our preliminary findings show MAMLD1 was found to be strongly associated to PA. While a relationship between gonadotroph pituitary adenomas and MAMLD1 has been elucidated [51], little is known about the function of MAMLD1 - to the best of our knowledge this is the first report link to Cushing’s disease. The Mastermind Like (MAML) family of genes vary significantly in function. MAMLD1 has a transcriptional co-activator role in regulating NOTCH signaling [39–41, 52]. Recently, another MAML family gene, Mastermind Like Transcriptional Coactivator 3 (MAML3) has been found to be associated with hereditary paragangliomas (PGL), a type of neuroendocrine tumor that is usually derived from extra-adrenal chromaffin cells as opposed to the pituitary [53]. However, despite being in the same family, MAML3 functions as a fusion protein, whereas MAMLD1 functions as an inhibitor protein. Evidence suggests MAMLD1 may bind the recombining binding protein suppressor of hairless (RBJP) repressor protein and subsequently inhibit hairy and enhancer of split gene (HES) expression [54]. During neuronal development, HES family members play a role in corticotrophic proliferation and differentiation [55], which HES1 knockouts has lead to an underdeveloped hypermorphic pituitary [56]. Our study underscores the role MAML proteins seem to play in regulating neuroendocrine function and encourages further functional investigations.

There are several limitations to this study. First, due to the rare quantity of corticotrophic PA, only four of the eight significantly overexpressed cross-species genes in RNA-seq were able to have been randomly selected and stained for IHC from archival specimens. Although a sensitivity analysis showed no difference, continued IHC studies should be performed for non-stained genes, for MAMLD1 replication in dog PA specimens, and for exploring the value of the commonly underexpressed genes. Second, while USP8 mutations where found in 40% of human PA (Caucasian and Asian populations), similar to other studies, neither of our cohorts (2.4% chance) found USP8 mutations or statistically significant DGE of POMC (due to wider expression variance) despite staining immuno-positive for well-established CD genes (POMC, TBX19) from intermediated sized PA female samples (African American population) [18–21]. Further exploration behind different populations and PA models may be of interest to fully illustrate the genetic variations of PA. Third, appropriate tissue was not available to confirm protein expression in the dog specimens and contrary to humans canine CD rise predominately from intermediate pituitary lobe as opposed to the anterior. However, 31 archival human PA provided strong evidence that co-overexpression of MAMLD1 and TBX19 occurred in approximately 70.9% of PA. Finally, although transcriptome analysis (Affymetrix arrays) on human PA has been complete [57, 58] and no functional MAMLD1 assays were complete, our current investigation was neither a discovery cohort for direct comparison nor a functional assessment study, respectively. Rather, our cohort aimed to broadly profile the somatic aberrations of PA between dog and man.

In spite of these genetic heterogeneities, differentially expressed genes were identified that were common between human and dog PA. Our study not only highlights and contributes to the growing complex understandings of genetic mechanism of CD, but also underscores the potential clinical implications of shared mechanisms between dog and man. Since MAMLD1 appears to stain exclusively for corticotrophic tumor cells as compared to TBX19 (may stain both normal and tumor corticotrophic cells), MAMLD1 staining may be helpful in diagnosing CD, particularly in microadenomas with aberrant in symptomology and potentially recurrent when tissue quantity is limited. This may improve CD clinical outcomes and help better characterize different hormone secreting pituitary tumors. Our results also suggests that, because MAMLD1 expression appears absent in normal pituitary, targeted inhibition of MAMLD1 may be a potential potent targeted strategy for inhibiting the growth of corticotroph PA that merits further functional exploration [7]. Furthermore, dog PA may provide suitable veterinary cohorts to test novel diagnostic and therapeutic approaches that may reduce CD burden in both dog and man.

## Conclusion

We highlight the first preliminary insights into profiling the genomic characterizations of human and dog corticotroph PA with respect to MAMLD1 overexpression, a finding of potential direct impact to CD microadenoma diagnosis. Our study also offers a rationale for potential use of the canine model in development of precision therapeutics.

## Supplementary Information

**Additional file 1: Table S1.** Post-agreement Immunohistochemistry Chemistry Scoring. Individualized immunohistochemistry scoring (0 to 3+) of Cushing’s pituitary adenoma human specimens from 7 antibodies (k = 0.87). **Fig. S1.** Difference in Magnitude of RNA-seq T-test Results. Normalized averaged FPKM values and standard errors are shown for genes suspected to be overexpressed by RNA-seq in human corticotroph pituitary adenomas (HC PA, *n* = 6) and dog corticotroph pituitary adenomas (DC PA, *n* = 7). **Fig. S2.** GeneCards® Summary Characteristics of Highly Expressed Genes in Humans and Dogs with Cushing’s disease. **Fig. S3.** Theoretical Schematic Representation of the Highly Expressed Genes in Humans and Dogs with Cushing’s disease.

## Data Availability

We are currently analyzing the data from a different perspective and planning a related study. Therefore, the data and material are not shared in the current state. However, the datasets used and/or analyzed during the current study are available from the corresponding author on reasonable written request. After the conclusion of the final study, all sequencing data will be made available in NCBI SRA database.

## Abbreviations

CD: Cushing’s Disease
PA: pituitary adenomas
MAMLD1: Mastermind Like Domain Containing 1
WES: Whole Exome Sequencing
RNA-Seq: Whole transcriptome shotgun sequencing
IHC: Immunohistochemistry
