# Supplementary material for: The genomic profiling and MAMLD1 expression in human and canines with Cushing’s disease
Source: BMC Endocr Disord. 2021 Sep 13;21:185. doi: 10.1186/s12902-021-00845-z (PMC8438999; doi:10.1186/s12902-021-00845-z)
Supplement: Supplementary file 1 — Additional file 1: Table S1. Post-agreement Immunohistochemistry Chemistry Scoring. Individualized immunohistochemistry scoring (0 to 3+) of Cushing’s pituitary adenoma human specimens from 7 antibodies (k = 0.87). Fig. S1. Difference in Magnitude of RNA-seq T-test Results. Normalized averaged FPKM values and standard errors are shown for genes suspected to be overexpressed by RNA-seq in human corticotroph pituitary adenomas (HC PA, n = 6) and dog corticotroph pituitary adenomas (DC PA, n = 7). Fig. S2. GeneCards® Summary Characteristics of Highly Expressed Genes in Humans and Dogs with Cushing’s disease. Fig. S3. Theoretical Schematic Representation of the Highly Expressed Genes in Humans and Dogs with Cushing’s disease. [file 12902_2021_845_MOESM1_ESM.docx]

**Supplementary:**

**SP Table. 1**

| **ID** | **MAMLD1** | **MNX1** | **RASEF** | **USP8** | **USP48** | **TBX 19** | **POMC** |
| --- | --- | --- | --- | --- | --- | --- | --- |
| **Negative Control** | 0 | 0 | 0 | 0 | 0 | 0 | 0 |
| **1** | 2 | 1 | 0 | 0 | 0 | 3 | 3 |
| **2** | 1 | 1 | 1 | 1 | 1 | 2 | 1 |
| **3** | 1 | 1 | 0 | 1 | 2 | 0 | 2 |
| **4** | 2 | 2 | 2 | 1 | 3 | 2 | 3 |
| **5** | 3 | 3 | 2 | 1 | 3 | 2 | 3 |
| **6** | 1 | 3 | 0 | 2 | 2 | 3 | 1 |
| **7** | 1 | 1 | 1 | 2 | 2 | 1 | 2 |
| **8** | 2 | 1 | 1 | 2 | 1 | 3 | 3 |
| **9** | 3 | 3 | 2 | 2 | 3 | 3 | 3 |
| **10** | 3 | 3 | 0 | 3 | 3 | 3 | 2 |
| **11** | 2 | 2 | 1 | 3 | 1 | 3 | 3 |
| **12** | 3 | 1 | 1 | 3 | 2 | 3 | 3 |
| **13** | 3 | 1 | 1 | 3 | 2 | 1 | 3 |
| **14** | 2 | 2 | 1 | 3 | 3 | 0 | 3 |
| **15** | 3 | 2 | 1 | 3 | 3 | 3 | 3 |
| **16** | 3 | 2 | 1 | 3 | 3 | 3 | 3 |
| **17** | 3 | 3 | 1 | 3 | 3 | 3 | 3 |
| **18** | 3 | 3 | 1 | 3 | 3 | 3 | 3 |
| **19** | 3 | 1 | 2 | 3 | 2 | 2 | 3 |
| **20** | 3 | 1 | 2 | 3 | 2 | 1 | 3 |
| **21** | 3 | 3 | 2 | 3 | 2 | 3 | 3 |
| **22** | 3 | 2 | 2 | 3 | 3 | 1 | 3 |
| **23** | 3 | 2 | 2 | 3 | 3 | 3 | 3 |
| **24** | 3 | 3 | 2 | 3 | 3 | 3 | 3 |
| **25** | 3 | 3 | 2 | 3 | 3 | 1 | 3 |
| **26** | 3 | 3 | 2 | 3 | 3 | 2 | 3 |
| **27** | 3 | 3 | 3 | 3 | 1 | 3 | 3 |
| **28** | 3 | 2 | 3 | 3 | 2 | 2 | 3 |
| **29** | 3 | 2 | 3 | 3 | 2 | 3 | 3 |
| **30** | 3 | 2 | 3 | 3 | 3 | 2 | 3 |
| **31** | 3 | 3 | 3 | 3 | 3 | 2 | 3 |

**Post-agreement Immunohistochemistry Chemistry Scoring.** Individualized immunohistochemistry scoring (0 to 3+) of Cushing’s pituitary adenoma human specimens from 7 antibodies (k=0.87).

**SP Fig. 1**


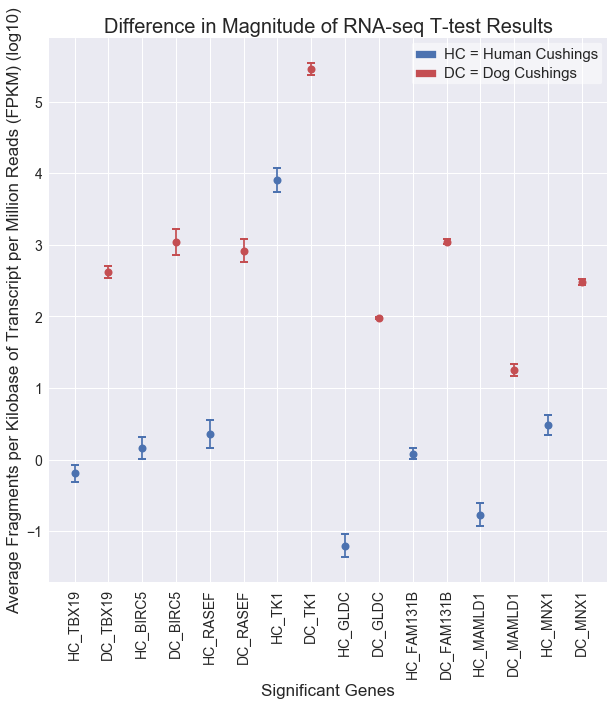


**Difference in Magnitude of RNA-seq T-test Results.** Normalized averaged FPKM values and standard errors are shown for genes suspected to be overexpressed by RNA-seq in human corticotroph pituitary adenomas (HC PA, n =6) and dog corticotroph pituitary adenomas  (DC PA, n=7).

**SP Fig. 2**

| **Gene Summary** | **AVPR1B** | **BIRC5** | **CEP55** | **FAM131B** | **FZD9** | **GLDC** | **MAMLD1** |
| --- | --- | --- | --- | --- | --- | --- | --- |
| **Chromosomal Location** | 1 | 17 | 10 | 7 | 7 | 9 | X |
| **Gene Function** | Receptor for Arginine Vasopressin | Inhibitor of Apoptosis (IAP) | Cytokinesis | MAPK Signaling | Receptor for WNT2 coupled with Beta-Catenin Canonical Signalling | Glycine Cleavage System | Transcriptional Co-Activator |
| **Primary Associated Disease(s)** | Bronchial Neuroendocrine Tumor, Acth-Independent Macronodular Adrenal Hyperplasia. | Adenocarcinoma, Cervical Intraepithelial Neoplasia, Vulvar Intraepithelial Neoplasia, Malignant Peritoneal Mesothelioma, Cervical Carcinosarcoma | Multinucleated Neurons, Anhydramnios, Renal Dysplasia, Cerebellar Hypoplasia, Hydranencephaly, Meckel Syndrome (Type 1) | Pilocytic Astrocytoma of Cerebellum, Cerebellar Astrocytoma | Williams Syndrome | Glycine Encephalopathy, Epilepsy (Idiopathic Generalized). | Hypospadias 2 (X-Linked), Posterior Hypospadias. |

| **Gene Summary** | **MNX1** | **POMC** | **PTGER4** | **RASEF** | **TBX19** | **TK1** | **VIPR2** |
| --- | --- | --- | --- | --- | --- | --- | --- |
| **Chromosomal Location** | 7 | 2 | 5 | 9 | 1 | 17 | 7 |
| **Gene Function** | Transcription Factor | Preproprotein | Receptor for Eicosanoid Ligand-Binding, GPCR Signaling | GTPases Involved in Membrane Trafficking | Transcription Factor | Cytosolic Enzyme (Adds Gamma-Phosphate Group to Thymidine) | Receptor for Vasoactive Intestinal Peptide |
| **Primary Associated Disease(s)** | Currarino Syndrome, Anorectal Anomalie | Obesity (Early-Onset with Adrenal Insufficiency and Red Hair), Body Mass Index Quantitative Trait Locus 11 | Malignant Epithelial Mesothelioma, Penis Basal Cell Carcinoma | Various Oncological Tumors Related to Tumor Suppressor Dysfunction | Acth Deficiency (Isolated, Late-Onset Isolated) | Hypochondroplasia, Thanatophoric Dysplasia (Type I) | Schizophrenia (16), Holoprosencephaly (3) |

**GeneCards ® Summary Characteristics of Highly Expressed Genes in Humans and Dogs with Cushing’s disease**.

**SP Fig. 3**

**
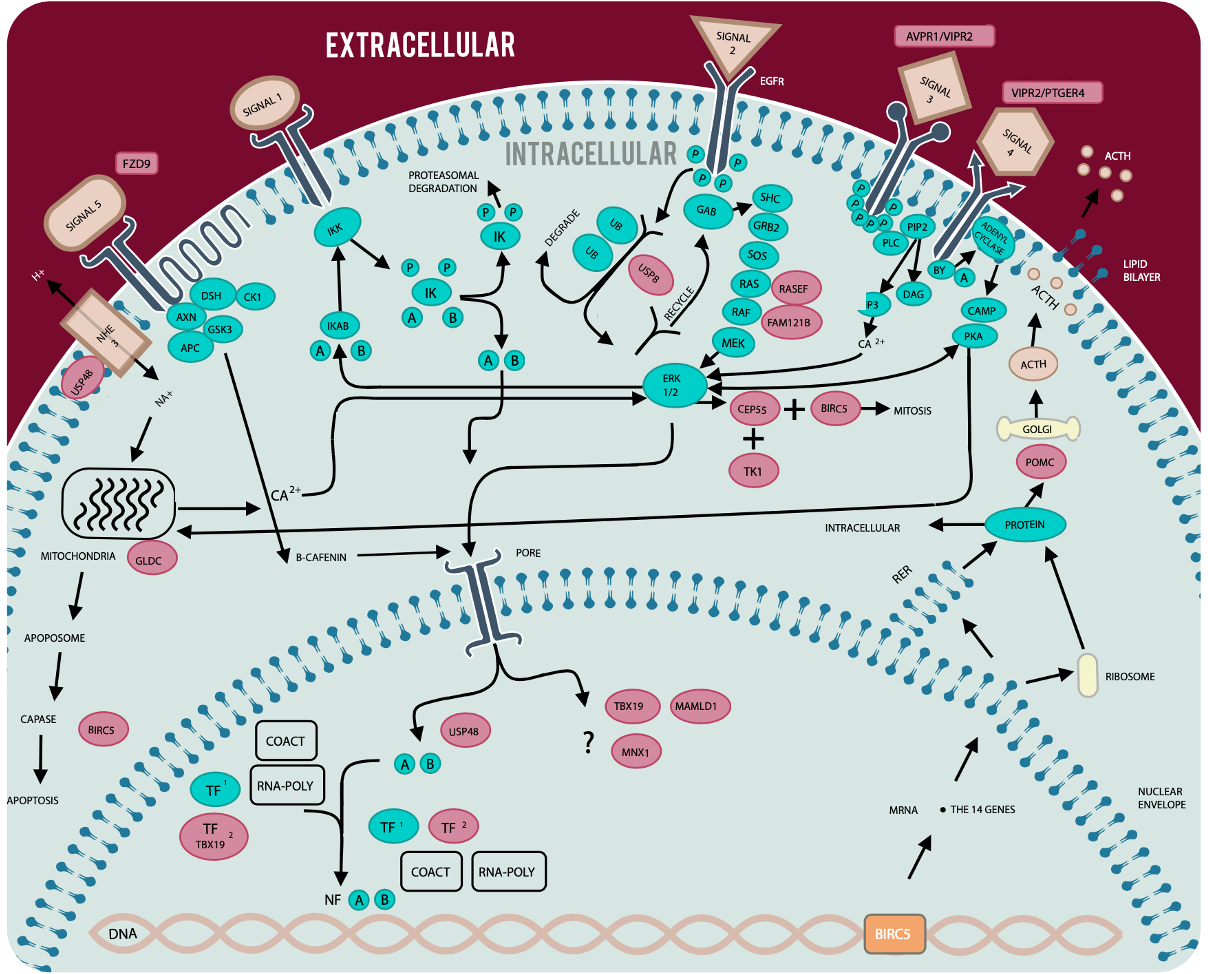
**

**Theoretical Schematic Representation of the Highly Expressed Genes in Humans and Dogs with Cushing’s disease.**
